# Association Between Liquid Biopsy and Prognosis of Gastric Cancer Patients: A Systematic Review and Meta-Analysis

**DOI:** 10.3389/fonc.2019.01222

**Published:** 2019-11-26

**Authors:** Yunhe Gao, Hongqing Xi, Bo Wei, Jianxin Cui, Kecheng Zhang, Hua Li, Aizhen Cai, Weishen Shen, Jiyang Li, Rafael Rosell, Joseph Chao, Tianhui Chen, Samuel Klempner, Zhi Qiao, Lin Chen

**Affiliations:** ^1^Department of General Surgery, Chinese PLA General Hospital, Beijing, China; ^2^General Surgery Institute, Chinese PLA General Hospital, Beijing, China; ^3^Nanjing General Hospital of Nanjing Military Command, Nanjing, China; ^4^Catalan Institute of Oncology, Germans Trias i Pujol Health Science Institute and Hospital, Barcelona, Spain; ^5^Department of Medical Oncology and Therapeutics Research, City of Hope Comprehensive Cancer Center, Duarte, CA, United States; ^6^Department of Cancer Prevention, Institute of Cancer and Basic Medicine (ICBM), Zhejiang Provincial Office for Cancer Prevention and Control, Cancer Hospital of the University of CAS, Chinese Academy of Sciences (CAS), Hangzhou, China; ^7^The Angeles Clinic and Research Institute, Los Angeles, CA, United States; ^8^Samuel Oschin Comprehensive Cancer Institute, Cedars-Sinai Medical Center, Los Angeles, CA, United States

**Keywords:** liquid biopsy, circulating tumor cells, circulating tumor DNA, circulating mRNA, gastric cancer, prognosis

## Abstract

**Background:** Reports regarding liquid biopsy and gastric cancer (GC) have emerged rapidly in recent decades, yet their prognostic value still remains controversial. This study was aimed to assess the impact of liquid biopsy, including circulating tumor cells (CTCs) and cell-free nucleic acids, on GC patients' prognosis.

**Methods:** PubMed, Medline, EMBASE, and ClinicalTrial.gov databases were searched for studies that report GC patient survival data stratified by CTC/circulating tumor DNA (ctDNA)/circulating miRNAs' status. The hazard ratios (HRs) and their 95% confidence intervals (CIs) for patients' overall survival (OS) and disease-free survival (DFS)/progression-free survival (PFS) were recorded or calculated depending on circulating target status.

**Results:** We initially identified 4,221 studies, from which 43 were eligible for further analysis, comprising 3,814 GC patients. Pooled analyses showed that detection of certain CTCs, ctDNA, and circulating miRNA was associated with poorer OS (CTCs: HR = 1.84, 95%CI 1.50–2.26, *p* < 0.001; ctDNA: HR = 1.78, 95%CI 1.36–2.34, *p* < 0.001; circulating miRNA: HR = 1.74, 95%CI 1.13–2.69, *p* < 0.001) and DFS/PFS (CTCs: HR = 3.39, 95%CI 2.21–5.20, *p* < 0.001; ctDNA: HR = 2.38, 95%CI 1.31–4.32, *p* = 0.004; circulating miRNA: HR = 3.30, 95%CI 2.39–4.55, *p* < 0.001) of GC patients, regardless of disease stage and time point at which sample is taken (at baseline or post-treatment).

**Conclusions:** The presence of CTCs and/or cellular components identifies a group of GC with poorer prognosis. Among circulating markers, CTCs demonstrated a stronger and more stable predictive value for late-stage disease and among Mongolian populations with GC. Less data are available for ctDNA and miRNA; however, their presence may also reflect aggressive biology and warrants further prospective study.

## Introduction

Gastric cancer (GC) remains the fifth most common cancer and the third leading cause of cancer-related death worldwide (1, 2). Although some therapeutic advances have been made, its prognosis remains unfavorable owing to the aggressive tumor biology, late detection, and high disease progression/recurrence rate (3). Few clinicopathological factors are used to guide therapy or disease monitoring, and ideal peripheral blood biomarkers have been lacking. Although enhanced endoscopic techniques, such chromoendoscopy (4) and endoscopy with narrow-band imaging (NBI) (5), are considered to be the more reliable and credible methods for diagnosis of GC than conventional diagnostic tools, their applications are limited because of their invasive nature and cost-efficacy concerns (5).

Although serum-based protein biomarkers such as carcinoembryonic antigen (CEA) (6), carcinoma antigen 125 (CA-125) (7), carcinoma antigen 724 (CA-724) (8), and carcinoma antigen 19-9 have commonly been used for GC patient management, they are plagued by limited diagnostic, and prognostic capacity (9). Circulating tumor cells (CTCs) and cell-free nucleic acids (cfNAs), known as “liquid biopsies,” are detectable biomarkers across tumor types and represent attractive putative targets in GC (10–13). The potential advantages of liquid biopsy have been demonstrated in the management of breast cancer, colorectal cancer, and prostate cancer (14–16), but evidence of their effectiveness in GC management is limited and controversial.

Theoretically, tumor-derived blood-based biomarker tests have multiple application in GC including detecting/monitoring response after therapies, identification of actionable tumor alterations, and patient stratification (17, 18). Currently, the diagnostic value of liquid biopsy is still under debate, and it has been questioned for its low sensitivity and yields in some series (12, 19). In contrast, the prognostic importance is increasingly supported by mounting evidence in breast (20) and colorectal (21) cancers. Although cfNAs include several cellular components, the most commonly investigated in GC research are circulating tumor deoxyribonucleic acid (ctDNA) (22) and circulating microRNA (miRNA) (23). Variability in detection methodology, genomic coverage, specimen processing, and reproducibility has not always been consistent. Moreover, the most appropriate sampling time point for accurate detection (at baseline or post-treatment), the most appropriate test population and disease stage, and even the predictive value of certain types of biomarkers have not yet been agreed (12, 24). With the continuously emerging data in GC, there is a need to conduct quantitative analysis evaluating the most commonly used liquid biopsy methods currently in GC management. Therefore, we sought to conduct a systematic review and meta-analysis to evaluate the significance of CTCs and cfNAs in predicting GC progression and recurrence in a methodologically consistent manner.

## Methods

### Literature Search

MOOSE (Meta-analysis Of Observational Studies in Epidemiology) (25) and PRISMA (Preferred Reporting Items for Systematic Reviews and Meta-Analyses) (26) guidelines were applied to conduct the systematic review. The following databases were systematically searched for relevant studies published up to December 2017: PubMed, Medline, EMBASE, Web of Science, and the Cochrane Central Register of Controlled Trials. Bibliographies of all relevant papers were also checked for further eligible studies. There was no restriction on language of publication (Table S1).

### Selection Criteria

Studies were included in the analysis if they met the following criteria: (1) they enrolled patients with pathologically confirmed gastric or gastroesophageal junction adenocarcinoma; (2) they reported GC patient survival data stratified by CTC/ctDNA/circulating miRNA status (presence/positive and absence/negative); (3) they provided sufficient data for determining or calculating a hazard ratio (HR) and 95% confidence interval (95%CI); and (4) they enrolled patients who did not overlap with patients included in other eligible studies.

Studies were excluded if (1) fewer than 20 patients were analyzed; (2) samples were not drawn from peripheral blood (e.g., from urine or bone marrow); or (3) the histology type of included GC patients was squamous carcinoma or neuroendocrine carcinoma.

### Data Extraction

Two authors (HX and JC) independently reviewed the eligible studies and extracted the following information: first author name, publication year, number of patients analyzed, age, gender, tumor stage, clinical treatment, volume and timing of blood withdrawal, marker detection method, cutoff value, positive ratio, and follow-up duration, if provided. When more than one marker was assessed in studies and an HR for survival or the survival curve was provided for each marker, results for all these markers were recorded as independent data sets.

### Assessment of Risk of Bias

Risk of bias for individual studies was assessed using a modified Cochrane risk of bias instrument that included evaluation options of “definitely or probably yes” or “definitely or probably no” or “unknown or unclear” (27). The items included “adequate eligibility,” “the measurement equality,” “controlled confounding,” “adequate follow-up,” “free of selective outcomes,” and “other factors” (Table S2) (21).

### Statistical Analysis

The HRs and their 95%CIs for overall survival (OS) and disease-free survival (DFS)/progression-free survival (PFS) were recorded. For studies where HRs were not provided, we approximated HRs from the Kaplan–Meier curves with the use of an HR calculation Excel spreadsheet provided by Tierney et al. (28). All HR data extraction and calculations were performed independently by YHG and HQX, and disagreements were resolved by discussion. Survival outcomes generated using multivariate analysis models were preferentially used if available, to ensure results are as clinically relevant as possible. By convention, an HR > 1 implies a worse prognosis in the circulating marker positive/upregulated group than in the negative/downregulated group, and *p* < 0.05 indicated statistical significance.

We pooled the extracted HRs using the generic inverse variance method. We anticipated interstudy heterogeneity and so used a random-effect analysis model preferentially (29). If no obvious heterogeneity was observed (*p* > 0.05), then a fixed-effect model was applied. Analyses were conducted using Stata 12.0 (StatCorp, College Station, TX, USA).

### Sensitivity Analysis, Subgroup Analysis, and Meta-Regression Analysis

The stability of pooled HRs was tested by one-way sensitivity analysis with omission of a single study. Subgroup analyses and meta-regression were performed to explore potential sources of heterogeneity, and the following clinicopathological features were stratified: sampling time (at baseline or postoperatively), number of tested targets, cutoff value, tumor–node–metastasis (TNM) stage, risk of bias level, statistical methodology employed, ethnicity, and sample size. Any subgroup comprising fewer than two studies was excluded from the analysis.

## Results

### Baseline Study Characteristics

**Figure 1:**
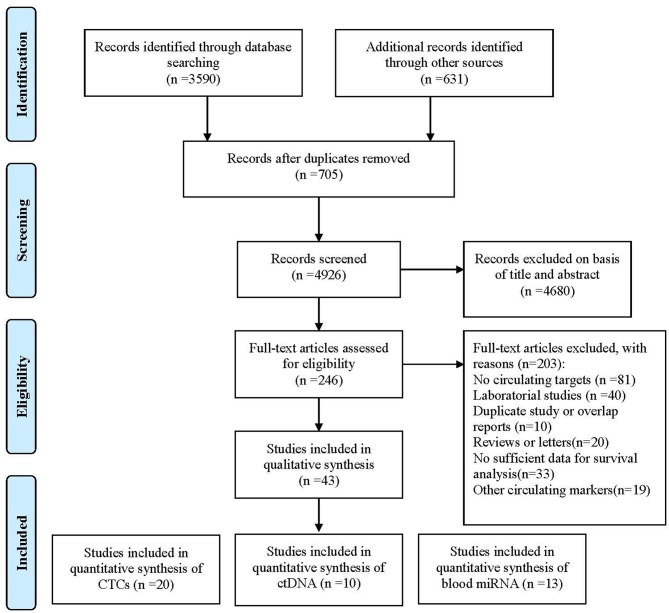
Flowchart of enrolled studies investigating the association of liquid biopsy and gastric cancer patients' prognosis.

Forty-three studies were eligible for inclusion, comprising 3,814 patients. These included 20 studies reporting on CTCs, 10 on ctDNA, and 13 on circulating miRNAs. Considering CTCs could also be performed at the DNA or RNA (mRNA or microRNA) level, we classified enrolled studies into relevant groups according to the authors' description in their report (Figure 1).

The baseline characteristics and study design variables of the included studies are shown in Table 1. All studies were written in English. Sample sizes ranged from 27 to 277 patients (median: 73 patients). The studies were conducted in 11 countries or regions (China, Egypt, Germany, Greece, Hong Kong, Italy, Japan, Poland, Spain, Taiwan, and Thailand).

**Table 1:** Baseline characteristics of included studies.

**Target**	**Detection method**	**References**	**Year**	**Number**	**M/F**	**Age**	**Region**	**Cancer stage**	**Treatment**	**Sample volume**	**Sample time**	**Positive ratio**	**Follow-up**	**Cutoff**	**HR estimate**	**Outcomes**	**Bias**
**CIRCULATING TUMOR CELLS**																	
CellSearch	CellSearch	Hiraiwa et al. (30)	2008	27	NR	68.9 ± 9.6	Japan	IV^m^	NR	7.5 ml	Baseline	15/27	5.8 (1.0–15.0)	2/7.5 ml	FC	OS	Low
CellSearch	CellSearch	Matsusaka et al. (31)	2010	52	44/8	62 (24–78)	Japan	IV^m^	Chemo	7.5 ml	AOT	17/52	/	4/7.5 ml	Provided (M)	PFS, OS	High
CellSearch	CellSearch	Uenosono et al. (32)	2013	148	99/49	57 (>70)	Japan	I–IV	Surgery (R0)	7.5 ml	Baseline	16/148	31.6 (4–72)	1/7.5 ml	Provided (M)	OS	Low
CellSearch	CellSearch	Li et al. (33)	2015	136	89/47	59 (25–80)	China	II–IV	Chemo	7.5 ml	Post-therapy	57/136	28.3 (median)	3/7.5 ml	Provided (M)	RFS, OS	Low
CellSearch	CellSearch	Okabe et al. (34)	2015	136	87/49	NR	Japan	II–IV	NR	7.5 ml	Baseline	25/136	26	1/7.5 ml	Provided (M)	PFS, OS	Low
Survivin	RT-PCR ELISA	Yie et al. (35)	2008	26	NR	NR	China	I–IV	Surgery (R0) + chemo	2 ml	Baseline	12/26	36	ROC	Provided (M)	RFS	High
Survivin	qRT-PCR	Bertazza et al. (36)	2009	70	39/31	68 (28–90)	Italy	I–IV	Surgery	6 ml	AOS	53/70	15 (6–119)	75th	Provided (M)	OS	Low
Survivin	RT-PCR ELISA	Cao et al. (37)	2011	98	63/35	NR	China	I–IV	Surgery	6 ml	Baseline	45/98	47.5 (36.5–56)	ROC	Provided (M)	DFS	Low
CK19	RT-PCR	Majima et al. (38)	2000	52	NR	NR	Japan	I–IV	Surgery	10 ml	Baseline	5/52	NR	HC	FC	OS	High
CK	FC+ IF	Noworolska et al. (39)	2007	57	44/13	NR	Poland	I–IV	Surgery + chemo	NR	Baseline	31/57	NR	3/slides	FC	OS	Low
CK20	RT-PCR	Illert et al. (40)	2005	70	48/22	69 (41–87)	Germany	I–IV	Surgery (R0 + R2)	9 ml	Baseline	28/70	20 (1–57)	HC	FC	OS	High
CK18/E-cadherin	qRT-PCR	Saad et al. (41)	2010	30	16/14	NR	Egypt	I–IV	Surgery + chemo	2 ml	Baseline	15/15	NR	HC	Provided (M)	OS, RFS	Low
CK	IF	Liu et al. (42)	2017	59	35/24	59 (median)	China	III–IV	Chemo	5 ml	Baseline	36/23	NR	2/5 ml^+^	Provided (M)	OS/DFS	Low
MUC1/C-Met	RT-PCR	Uen et al. (43)	2006	52	31/21	30 (>60)	Taiwan	I–IV	Surgery	5 ml	AOS	32/52 (C-met), 37/52 (MUC1)	NR	5/ml	FC	OS	High
hTERT/CK19/CEA/MUC1	CMA^3^	Wu et al. (44)	2006	64	41/23	60.5 (36–84)	Taiwan	I–IV	Surgery	4 ml	AOS	25/39	28 (20–33)	ROC	FC	OS/DFS	Low
CEA	RT-PCR	Ikeguchi et al. (45)	2005	59	38/21	66.3 (26–86)	Japan	I–IV	Surgery	1.5 ml	AOS	27/43	20.1 (2–31)	PC	FC	OS/DFS	High
CEA	qRT-PCR	Ishigami et al. (46)	2007	67	46/21	65 (median)	Japan	I–IV	Surgery (R0)	5 ml	AOS	33/67	37 (23–48)	PC	FC	OS	High
CEA	RT-PCR	Qiu et al. (47)	2010	123	82/41	59 (28–84)	China	I–IV	Surgery (R0) + chemo	5 ml	Baseline	45/123	37 (3.0–73.6)	PC	Provided (M)	DFS	Low
B7-H3	RT-PCR	Arigami et al. (48)	2010	95	64/31	47 (>70)	Japan	I–IV	Surgery (R0)	5 ml	Baseline	48/95	24 (1–74)	ROC	Provided (M)	OS	Low
Telomerase	IF	Ito et al. (49)	2016	65	46/19	58.8 (33–76)	Japan	I–IV	Surgery (R0 + R1)	7.5 ml	Baseline	18/47	60	ROC	Provided (M)	OS/RFS	Low
**CIRCULATING TUMOR DNA**																	
APC/E-cadherin	MSP	Leung et al. (50)	2005	60	35/25	66 (35–96)	Hong Kong	I–IV	Surgery	NR	Baseline	7/53	8 (0–40)	HC	FC	OS	High
SOX17	MSP	Ioanna et al. (51)	2013	73	51/22	56 (>60)	Greece	NR	Surgery (R0)	NR	Baseline	43/30	56 (20–111)	HC	FC	OS	Low
BCL6B	BGS	Yang et al. (52)	2013	40	33/7	NR	China	IV	NR	1 ml	Baseline	17/23	NR	HC	FC	OS	High
XAF1	MSP	Ling et al. (53)	2013	202	120/87	57 (≥60)	China	I–IV	Surgery	NR	Baseline	141/61	NR	HC	FC	DFS	Low
MINT2	qMSP	Han et al. (54)	2014	92	53/39	24 (≥60)	China	I–IV	Surgery	NR	Baseline	36/56	NR	ROC	Provided (U)	DFS	Low
P16	qMSP	Wu et al. (55)	2014	92	53/39	24 (≥60)	China	I–IV	Surgery	NR	NR	63/29	NR	HC	FC	DFS	Low
TIMP−3	MSP	Yu et al. (56)	2014	92	54/38	24 (≥60)	China	I–IV	Surgery	NR	Baseline	54/38	NR	ROC	Provided (U)	PFS	Low
APC/ RASSF1A	MSP	Ioanna et al. (57)	2015	73	51/11	70.5 (28–82)	Greece	I–III	Surgery(R0)	NR	Baseline	61.50/73	56 (12–111)	HC	FC	OS	Low
PCDH10/ RASSF1A	MSP	Pimson et al. (58)	2016	101	44/57	30 (≥61)	Thailand	I–IV	NR	NR	NR	95.17/101	NR	HC	FC	OS	High
ARID1A/ P53/PIK3CA/ PTEN/AKT2	NGS	Fang et al. (59)	2016	277	212/65	174/277	Taiwan	I–IV	Surgery	NR	Baseline	138/139	61 (2–232)	Median	FC	OS	Low
**CIRCULATING microRNAs**																	
MiR-200c	RT-PCR	Ayerbes et al. (60)	2012	52	42/10	65.3 (49–74)	Spain	I–IV	Surgery + chemo	10 ml	AOS	28/24	24 (6–53)	ROC	Provided (M)	OS, PFS	Low
MiR-200c	qRT-PCR	Zhang et al. (61)	2015	98	53/45	51 (≥60)	China	I–IV	Surgery	5 ml	Baseline	50/48	NR	Median	Provided (M)	OS	Low
MiR-20a-5p	RT-qPCR	Yang et al. (62)	2017	55	35/20	33 (≥60)	China	I–IV	Surgery	4 ml	Baseline	27/28	NR	Median	FC	OS	High
MiR-20a	qRT-PCR	Wang et al. (63)	2012	65	34/31	44 (>60)	China	I–IV	Surgery	2 ml	Baseline	34/31	NR	Median	Provided (M)	OS	Low
MiR-21	qRT-PCR	Komatsu et al. (64)	2013	69	43/26	40 (>65)	China	I–IV	Surgery	7 ml	Baseline	47/22	NR	HC	Provided (M)	DFS	Low
MiR-21	qRT-PCR	Song et al. (65)	2013	103	68/35	60 (27–87)	China	I–IV	Surgery	5 ml	Baseline	51/52	35.9 (24.4–53.1)	Median	Provided (U)	OS	Low
MiR-206	RT-PCR	Hou et al. (66)	2016	150	98/52	59.8 (mean)	China	I–IV	Surgery (R0)	5 ml	Baseline	75/75	38	ROC	FC	OS, DFS	Low
Mir203	qRT-PCR	Imaoka et al. (67)	2016	130	122/61	66 (≥68)	Japan	I–IV	Surgery + chemo	5 ml	NR	53/77	31.4 (1–78)	ROC	FC	OS, DFS	Low
MiR-222	qRT-PCR	Fu et al. (68)	2014	114	54/60	46 (>50)	China	I–IV	NR	<8 ml	Baseline	75/39	24 (4–60)	ROC	Provided (M)	OS, DFS	Low
MiR-27a	qRT-PCR	Huang et al. (69)	2014	82	52/30	31 (>60)	China	IV	Chemo	NR	Baseline	41/41	NR	Median	Provided (M)	OS	Low
MiR-23b	qRT-PCR	Zhuang et al. (70)	2016	138	85/53	64 (≥60)	China	I–IV	NR	5 ml	Baseline	79/79	NR	Median	Provided (M)	OS, DFS	Low
MiR-196a/b	qRT-PCR	Tsai et al. (71)	2016	98	57/41	53 (≥65)	Taiwan	I–IV	Surgery	NR	Baseline/AOS	49/49	83 (64–137)	ROC	Provided (M)	OS,	Low
MiR-192/MiR-122	qRT-PCR	Chen et al. (72)	2013	72	54/18	57 (44–62)	China	III–IV	Chemo	3–5 ml	Baseline	34, 35/72	NR	ROC	Provided (M)	OS	Low
Total	43		3814														

*HC, healthy control; NC, negative control; PC, positive control; NR, not reported; FC, figure calculation; HR, hazard ratio; ROC, receiver operating characteristic curve; OS, overall survival; DFS, disease-free survival; PFS, progression-free survival; +, 5/ml KATO-III GC cell in healthy control volunteers; 3, colorimetric membrane array; MSP, methylation-specific PCR; BGS, bisulfite genomic sequence; qMSP, quantitative methylation-specific PCR; NGS, next generation sequencing; U, univariate; M, multivariate; R0, radical resection; m, metastatic cancers*.

All 43 studies applied a molecular or cytological detection method analyzing venous blood [polymerase chain reaction (PCR), quantitative reverse PCR (qRT-PCR), methylation-specific PCR (MSP), quantitative MSP (qMSP), next-generation sequencing (NGS), immunofluorescence (IF), CellSearch System, or colorimetric membrane array (CMA)]. Notably, three studies applied a combination of molecular and cytological detection methods (35, 37, 39). Five studies (37, 43, 57, 71, 72) analyzed the same patient cohort but using two different targets. To account for this, both markers were included in the pooled analysis, whereas the total number of patients was only counted once. The assessment of risk of bias for individual studies showed 31 and 12 studies with a low risk of bias and a high risk of bias, respectively. HRs for OS and DFS/relapse-free survival (RFS) could be extracted from 35 to 16 studies, respectively. Publication bias analyses were carried out for the analysis of all studies in Egger's and Begg's tests on OS and DFS/RFS, but no relevant publication bias was observed (Figure S1).

### Circulating Tumor Cells

**Figure 2:**
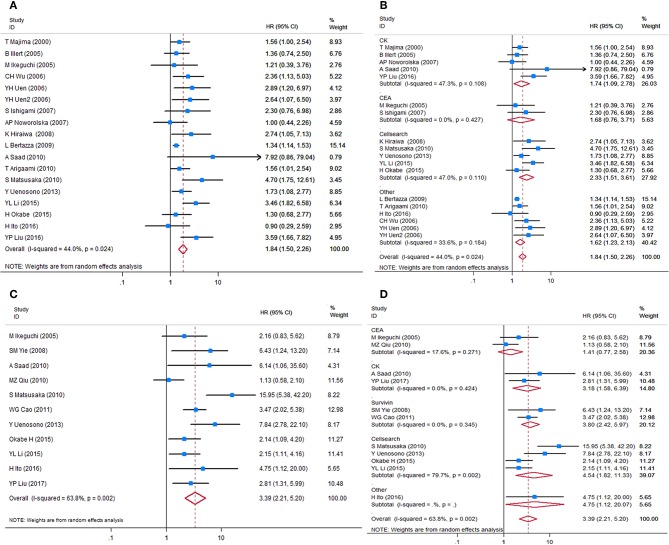
Forest plots of HRs for OS and DFS/PFS of GC patients, by CTC status. **(A)** Overall analysis of HR for OS of GC patients. **(B)** Subgroup analysis of HR for OS of the GC patients by detection targets. **(C)** Overall analysis of HR for DFS/PFS of GC patients. **(D)** Subgroup analysis of HR for DFS/PFS of GC patients by detection targets. HRs, hazard ratios; OS, overall survival; DFS, disease-free survival; PFS, progression-free survival; GC, gastric cancer; CTC, circulating tumor cell.

HRs for OS were available in 17 studies, representing 1,239 patients. Two HR estimates for OS were extracted from Uen et al. for the reason mentioned in the *Methods* part (43). The pooled HR showed a significant prognostic effect of CTC detection in GC patients (HR = 1.84, 95%CI 1.50–2.26, *p* < 0.001, Figure 2A), with moderate heterogeneity (*I*^2^ = 44%, *p* = 0.024).

HRs for DFS/PFS were available in 11 studies, representing 848 patients. The pooled HR showed a significantly increased risk of disease progression or recurrence in patients with CTC positivity (HR = 3.39, 95%CI 2.21–5.20, *p* < 0.001). The heterogeneity between studies was significant (*I*^2^ = 63.8%, *p* = 0.002).

Sensitivity analyses conducted by omitting each single study changed this result only marginally (Figure S2). Table 2 shows the results of subgroup analysis stratified by covariates of clinical importance as described in the Methods. The most popular applied markers were CellSearch-associated [a combination of cytokeratins and epithelial cell adhesion molecule (EpCAM)] cytokeratins and survivin, and a subgroup analysis based on CTC markers showed that all CTC markers were significantly associated with GC patients' OS and DFS/PFS, except for CEA (OS: HR = 1.68, 95%CI 0.76–3.71, *p* = 0.20; DFS/PFS: HR = 1.41, 95%CI 0.77–2.55, *p* = 0.262) (Figures 2B–D). There was a more pronounced predictive value for CellSearch in both OS and DFS/PFS prediction (OS: HR = 2.33, 95%CI 1.51–3.61; DFS/PFS: HR = 4.54, 95%CI 1.82–11.33) than other CTC detection markers. However, this observation could not be substantiated by further statistical tests of interaction.

**Table 2:** Subgroup analyses and meta-regression analyses.

	**CTCs**								**miRNA**				**ctDNA**			
	**OS**				**DFS**				**OS**				**OS**			
	***N***	**HR (95%CI)**	***I*^**2**^ (%)**	***P*^**m**^**	***N***	**HR (95%CI)**	***I*^**2**^ (%)**	***P*^**m**^**	***N***	**HR (95%CI)**	***I*^**2**^ (%)**	***P*^**m**^**	***N***	**HR (95%CI)**	***I*^**2**^ (%)**	***P*^**m**^**
**Sampling time**																
Baseline	10	1.63 (1.30–2.04)	13.3	0.225	8	3.15 (1.99–5.0)	56.1	0.755	2	2.58 (1.55–4.31)	0	0.9	/	/	/	/
Post-op	8	2.30 (1.52–3.49)	64.4		3	4.04 (1.21–13.44)	82.4		9	2.04 (1.45–2.88)	63.9	/	/	/	/	/
**Cutoff**																
HC/NC	5	1.57 (1.13–2.19)	0	0.267	3	1.86 (0.84–4.13)	46.6	0.278		/	/	/	5	1.94 (1.35–2.78)	49.7	0.520
ROC	3	1.62 (1.10–2.39)	5.8		3	3.88 (2.52–5.97)	0.00		5	2.60 (1.83–3.71)	15.3	0.275	/	/	/	/
Percentiles	9	2.27 (1.60–3.22)	67.0		5	4.02 (2.00–8.10)	73.4		6	1.86 (1.26–2.75)	63.0		1	1.78 (1.36–2.34)	/	/
**TNM stage**																
With early stage	13	1.45 (1.28–1.63)	0.2	0.01	7	3.38 (1.91–5.99)	61.4	0.437	9	2.32 (1.67–3.23)	57.7	0.268	5	1.79 (1.28–2.49)	58.8	0.865
Advanced or late stage	5	2.81 (1.79–4.40)	38.0		4	3.49 (1.63–7.53)	75.2		2	1.53 (0.97–2.41)	0		2	1.92 (1.15–3.20)	0	/
**Risk bias**																
Low	11	1.84 (1.50–2.26)	52.1	0.689	6	2.87 (1.58–5.24)	60.0	0.497	2	2.34 (1.37–3.98)	0	0.27	3	1.52 (1.14–2.05)	41.	0.148
High	7	1.96 (1.36–2.82)	21.3		5	4.06 (2.15–7.68)	67.6		9	2.11 (1.51–5.94)	61.0		3	2.33 (1.56–3.50)	17.5	
**Ethnicity**																
Caucasian	4	1.34 (1.16–1.54)	0	0.008	1	6.14 (1.06–35.58)	/	0.600	1	2.24 (1.09–4.61)	/	/	4	1.92 (1.38–2.67)	45.3	0.465
Mongolian	14	2.04 (1.64–2.54)	20.8		10	3.31 (2.12–5.16)	66.6		10	2.13 (1.57–2.90)	57.4		2	1.55 (0.85–2.83)	54.2	
**Sample size**																
<100	14	1.84 (1.45–2.33)	44.5	0.955	6	4.95 (2.92–8.37)	43.0	0.075	7	2.04 (1.56–2.67)	25.3	0.14	4	1.81 (1.25–2.62)	42.3	0.975
≥100	4	1.86 (1.19–2.92)	44.0		5	2.25(1.32–3.86)	59.1		4	2.27 (1.08–4.76)	53.4)		2	1.81 (1.11–2.95)	66.2	

*HR, hazard ratio; P^h^, p value for inter-study heterogeneity; P^m^, p value for meta-regression; HC, healthy control; NC, negative control; ROC, receiver operating characteristic curve*.

Meta-regression identified cancer stage and patient ethnicity as variables influencing OS HR estimates for CTCs (Table 2, *p* = 0.010 and *p* = 0.008, respectively). The presence of CTCs is associated with a higher HR for OS in studies enrolling only late-stage patients (HR = 2.81, 95%CI 1.79–4.40, *p* < 0.001) than studies enrolling with both early- and late-stage patients (HR = 1.84, 95%CI 1.50–2.26, *p* < 0.001). Nevertheless, both results from subgroups by TNM stage indicated a significant association between CTCs presence and worse prognosis of GC patients.

Studies involving GC patients of Mongolian ethnicity had a significantly higher pooled HR (2.04, 95%CI 1.64–2.54, *p* < 0.001) than had studies involving Caucasian patients (HR = 1.34, 95%CI 1.16–1.54, *p* < 0.001). This was further supported by tests for interaction (*p* = 0.008, Table 2). However, these differences by disease stage and ethnicity found in a subgroup analysis of OS HRs were absent in the analysis of DFS/PFS (Table 2). No other variables were found to be significant, which may be because of the relatively limited number of studies reporting DFS/PFS (11 studies in total, only one of which studied a primarily Caucasian patient population).

The subgroup analysis on sampling time showed a prognostic effect of CTC detection for both time points (baseline and during/post-treatment). HRs for CTCs predicting the survival of GC patients where liquid biopsies were taken during/post-treatment were higher than HRs of patients where biopsies were taken at baseline. This was the case for both OS (HR = 2.30, 95%CI 1.52–3.49, *p* < 0.001 during/post-treatment; HR = 1.63, 95%CI 1.30–2.04, *p* < 0.001 at baseline) and DFS/PFS (HR = 4.04, 95%CI 1.21–13.44, *p* = 0.023 during/post-treatment; HR = 3.15, 95%CI 1.99–5.0, *p* < 0.001 at baseline). However, this difference did not reach statistical significance and could not be substantiated by further tests of interaction.

### Circulating Tumor DNA

**Figure 3:**
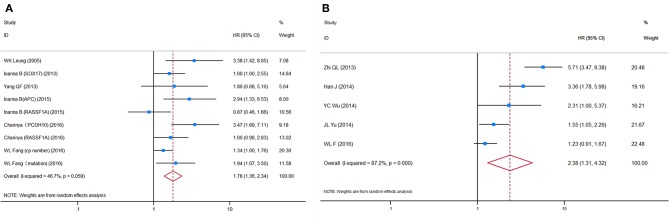
Forest plots of HRs for **(A)** OS and **(B)** DFS/PFS of GC patients, based on detection of circulating tumor DNA status. HRs, hazard ratios; OS, overall survival; DFS, disease-free survival; PFS, progression-free survival; GC, gastric cancer.

HRs for OS were reported in six studies, representing 624 patients. More than one HR for OS was extracted from three studies, because multiple detection approaches were used. The pooled HRs showed a significant prognostic effect of the detection of ctDNA in GC patients' OS (HR = 1.78, 95%CI 1.36–2.34, *p* < 0.001, Figure 3A), with moderate heterogeneity (*I*^2^ = 46.7%, *p* = 0.059). No ctDNA targets were assessed by more than two independent studies. Therefore, a subgroup analysis by target was not performed. A subgroup analysis by other variables revealed that ctDNA presence was significantly associated with shorter survival for all subgroups except in studies conducted primarily in Caucasian patients (*N* = 2, HR = 1.55, 95%CI 0.85–2.83, *p* = 0.156). However, this result must be interpreted with caution, given the small sample size. A Galbraith plot indicated that the study by Pimson et al. (58) might be one important source of heterogeneity (Figure S3A). Exclusion of Pimson et al. focusing on PCDH10 resulted in a significant decrease in heterogeneity (*I*^2^ = 33.2%, *p* = 0.163), but the association between ctDNA and OS remained significant (HR = 1.64, 95%CI 1.28–2.10, *p* < 0.001).

HRs for DFS/PFS were reported by five studies utilizing ctDNA, representing 731 patients. The pooled HR showed a significantly increased risk of disease progression or recurrence in patients with ctDNA detection (HR = 2.38, 95%CI 1.31–4.32, *p* = 0.004). Heterogeneity between studies was significant (*I*^2^ = 87.2%, *p* < 0.001). A Galbraith plot and a sensitivity analysis were performed to explore the source of heterogeneity and stability of the results. Although a sensitivity analysis showed that omission of any single study would not substantially alter the outcomes, the Galbraith plot showed that Fang et al. (59) and Ling et al. (53) were outliers and the main contributors to heterogeneity (Figure S3B). Excluding these two studies reduced heterogeneity somewhat (*I*^2^ = 56.5%, *p* = 0.100) and made the association between ctDNA and DFS/PFS more significant (HR = 2.19, 95%CI 1.31–3.66, *p* = 0.003, Figure 3B).

### Circulating miRNA

**Figure 4:**
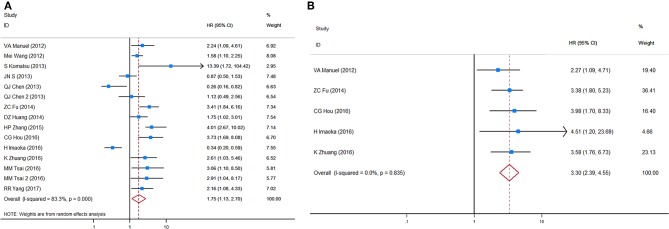
Forest plots of HRs for **(A)** OS and **(B)** DFS/PFS of GC patients, based on detection of circulating miRNA status. HRs, hazard ratios; OS, overall survival; DFS, disease-free survival; PFS, progression-free survival; GC, gastric cancer.

HRs for OS in circulating miRNA were available in 13 studies, representing 1,157 patients, and indicated a prognostic effect of circulating miRNA detection (HR = 1.75, 95%CI 1.13–2.70, *p* < 0.001), and there was considerable heterogeneity (*I*^2^ = 83.3%, *p* = 0.000) (Figure 4A).

After sensitivity analyses were performed, it was found that by excluding the only two studies with an HR estimate <1 (67, 72), the adjusted pooled HR for OS was higher (HR = 2.13, 95%CI 1.61–2.83, *p* < 0.001) whereas heterogeneity was substantially reduced (*I*^2^ = 53.4%, *p* = 0.014). In a manual review of the original work of these two studies, the authors considered the two targets, miR-203 and miR-122, as anti-tumor microRNAs on the basis of biological function. Therefore, a subgroup analysis and a meta-regression analysis were performed after excluding these targets, and then significant associations between circulating miRNA detection and OS were found in both sample time point groups (baseline and during/post-treatment). Unlike CTC analyses, a subgroup analysis stratified by tumor stage (all stages vs. advanced stage only) and ethnicity (Caucasian vs. Mongolian) did not alter the bias and differences significantly between these subgroups.

HRs for DFS/PFS in circulating miRNA were available in five studies, representing 584 patients. The pooled HR for DFS/PFS was 3.30 (95%CI 2.39–4.55, *p* < 0.001, Figure 4B). Heterogeneity between HR estimates was not significant (*I*^2^ = 0.0%, *p* = 0.835).

## Discussions

Here, we report the largest meta-analysis of circulating tumor-derived biomarkers and identify prognostic value for CTCs. Our meta-analysis provides strong evidence, even after adjustment for clinical variables. With over 3,800 included GC patients, our study is the most comprehensive systematic review of the association between liquid biopsy and GC prognosis to date, substantially larger than previous studies (73, 74).

Importantly, we attempted to address biomarker detection method, study heterogeneity, and disease stage. The association between biomarker detection (CTC, ctDNA, or miRNA) was relatively stable and not influenced largely by liquid biopsy detection methods or disease stage. Even among GC patients where samples were taken post-treatment, the association remained significant, highlighting the potential clinical utility of blood-based biomarkers in GC.

Overall, we observed a stronger association between circulating marker detection and DFS than OS, suggesting an important role in prognosis and patient stratification, particularly in non-metastatic patients. We acknowledge that an optimal cutoff value for each detection method remains to be determined, and a decreased heterogeneity was observed in the receiver operating characteristic (ROC) cutoff determination subgroups, indicating more consistent results in studies that adapted ROC curves to determine patients' tumor status.

Among the detection platforms examined, several important observations warrant further discussion, and the analysis of whole CTCs can be performed at the DNA or RNA (mRNA or microRNA) and protein levels, whereas the analysis of ctDNA and microRNAs can be performed only at the genomic level. For example, one alternative to enumerating CTCs by immunocytochemistry (ICC) is to estimate their presence using RT-PCR to discover epithelial transcripts, which should not be present in normal hematopoietic cells. However, detection of CTCs often requires more cumbersome enrichment and detection methods, whereas the detection of cfNAs can be performed using blood plasma or serum, and easier methods (24). Huang et al. (73) and our previous report (75) have demonstrated the significance of CTC and ctDNA in GC patients' prognosis prediction.

Among studies examining CTCs, the CellSearch System was the most widely used method for detecting and enumerating CTCs from blood samples, using a combination of epithelial markers (EpCAM+; cytokeratin 8, 18, and/or 19; and CD45–). It is still the first and only actionable commercial test for detecting CTCs in cancer patients, including metastatic breast (14), prostate (15), and colorectal cancers (16). Our results further support its application for GC patients as a statistically significant predictor of shorter OS and DFS/PFS.

Another popular marker type in CTC detection was found to be cytokeratins (CKs). CKs have been found to have different predictive values in patients from Asian (*N* = 2, HR = 3.54, 95%CI 1.84–6.82, *p* < 0.001) and Western populations (*N* = 3, HR = 1.38, 95%CI 0.73–2.61, *p* = 0.328), which suggests that they may play a different role in different ethnicities. Moreover, CKs have tended to serve as biomarkers in a combination of their own components (e.g., CK18, CK19, and CK20) (76) or alongside other targets such as EpCAM (77, 78) to identify the epithelial cells more precisely.

Circulating tumor DNA is composed of small fragments of nucleic acid that are not associated with cells or cell fragments (79). The most widely used method of detection is methylated DNA in plasma/serum, which is usually identified by MSP or quantitative MSP (qPCR) assays (80). All included studies withdrew blood for ctDNA detection at the baseline time point, which is probably because ctDNA is rapidly cleared from circulation after surgery or other therapy because of its short half-life (80). However, a previous study reported that DNA methylation is relatively chemically stable and can be detected at a sensitivity of up to 1:1,000 molecules (81). It is therefore not surprising that most of the studies included in our review focused on epigenetic regulation of circulating markers. Only Fang et al. investigated the role of gene mutation and copy number.

Although the dysregulation of ctDNA is relatively common in gastroesophageal cancers (22), a reliably detectable prognostic ctDNAs with high specificity is yet to be identified. Our meta-analysis only covers genes and epigenetic regulators relevant to GC. Whole gene screening assays, especially for genetic mutations, are required to identify more associations (82, 83).

Beyond CTCs and ctDNA, circulating miRNAs (miRNAs) are a large group of short, non-coding RNAs, 19–25 nucleotides long, which regulate gene expression by pairing to the 3′ untranslated region (3′-UTR) of their target mRNA (84). It has been suggested that miRNAs could function as either tumor suppressor or oncogenes by regulating gene expression at transcriptional and translational levels in GC (85). Notably, although not all detected CTCs are predictors of adverse outcomes (86), the majority of them are. In contrast, certain miRNAs detected in serum/plasma may be positive predictors of GC patient survival, acting as tumor-suppresser genes, such as miR-192 and miR-203. Nevertheless, our results also support previous evidence that oncogenic circulating miRNAs are strongly significant predictors of poorer outcomes, particularly for GC recurrence, and progression (HR = 3.41, 95%CI 2.48–4.69, *p* < 0.001; *I*^2^ = 0.0%, *p* = 0.670).

In the past, it has been difficult to obtain tumor samples from GC patients without surgery, as endoscopic biopsy provides limited genetic or cellular materials in most cases. Although the optimal platform remains open to debate, the ability of ctDNA to simultaneously detect genomic alterations is attractive and might have a prognostic role. Our meta-analysis supports the use of a series of detection targets and methods for predicting GC patients' OS and DFS. Intriguingly, our data suggest that detection of certain circulating markers at any time, pre-treatment, or post-treatment, provides important prognostic information. Among the overall advanced disease population, the presence of CTCs and tumor-related nucleic acids may help identify those patients that could benefit most, or at least, from systematic therapy including chemotherapy, target therapy, or immunotherapy (87, 88). In the era of NGS and a combination of multi-analytic biomarkers (89–91), our meta-analysis provides a solid foundation and methodological reference for further study.

We acknowledge several limitations to our large meta-analysis. First, studies may tend to selectively report their positive results, leading to risk of selection and publication bias. Second, the majority of our studies enrolled patients from all disease stages, making it difficult to stratify the prognostic value of circulating biomarkers by stage. In addition, a subgroup analysis of some variables involved groups with small sample sizes, which might bias our conclusions. Although meta-regression has indicated that tumor stage and ethnicity may contribute to inter-study heterogeneity in prognostic value, large, multicenter prospective studies based on homogeneous patient populations are still required to validate our findings.

## Conclusions

In conclusion, results of this meta-analysis demonstrated a significant role for liquid biopsy, including CTCs, ctDNA, and circulating miRNA, in predicting worse prognosis of patients with GC. By analyzing currently available studies, CTCs demonstrated a stronger and more stable predicative value in late-stage disease and Mongolian populations compared with early-stage disease and Caucasian populations, respectively. Careful selection of circulating markers and standard detection methods are likely to be fundamental to optimizing the accuracy of liquid biopsy in determining GC patients' prognosis. And further multicentered studies applying specific circulating biomarkers are warranted to clarify the clinical validity of liquid biopsy and its utility in GC patients.

## Author Contributions

YG, BW, and LC conceived the study. YG, HX, and JCu conducted the literature searches and extracted the data. AC, WS, and JL took part in the analysis and interpreted the data. YG, ZQ, and HX drafted the manuscript. RR, TC, JCh, and SK critically revised the manuscript. HL, JL, and ZQ helped in making the figures and tables. BW and KZ double-checked the extracted data.

### Conflict of Interest

The authors declare that the research was conducted in the absence of any commercial or financial relationships that could be construed as a potential conflict of interest.
