# Supplementary material for: Association Between Liquid Biopsy and Prognosis of Gastric Cancer Patients: A Systematic Review and Meta-Analysis
Source: Front Oncol. 2019 Nov 26;9:1222. doi: 10.3389/fonc.2019.01222 (PMC6901923; doi:10.3389/fonc.2019.01222)
Supplement: Supplementary file 1 [file Data_Sheet_1.pdf]

**Table S1. Search strategies**

**Pubmed:** ((((((((circulating tumor cell) OR blood tumor cells) OR serum tumor cell) OR plasma tumor cells)) OR ((circulating tumor dna) OR blood tumor dna) OR serum tumor dna) OR plasma tumor dna)) OR (((circulating mirna) OR blood mirna) OR serum mirna) OR plasma mirna))) AND ((gastric cancer) OR stomach cancer) OR gastric neoplasm) OR stomach neoplasm) OR gastroesophageal cancer) OR gastroesophageal tumor) OR gastric tumor) OR stomach tumor))) AND (((prognosis) OR hazard ratio) OR survival rate) OR hazard ratios) Filters: Publication data 2017/09/31.

**Embase:** ('circulating tumor cell' OR 'blood cancer cell' OR (serum AND tumor AND cell) OR (plasma AND tumor AND cell)) AND ('stomach cancer' OR 'gastric cancer' OR 'stomach neoplasm' OR 'gastric neoplasm' OR 'stomach tumor' OR 'gastric tumor') AND ('prognosis' OR 'hazard ratio' OR 'survival')

('circulating tumor dna' OR 'circulating tumor deoxyribonucleic acid' OR 'cell-free dna' OR 'blood tumor dna' OR (serum AND tumor AND dna) OR (plasma AND tumor AND dna)) AND ('stomach cancer' OR 'gastric cancer' OR 'stomach neoplasm' OR 'gastric neoplasm' OR 'stomach tumor' OR 'gastric tumor') AND ('prognosis' OR 'hazard ratio' OR 'survival')

('circulating tumor micro rna' OR 'circulating tumor mirna' OR 'blood tumor mirna' OR (serum AND tumor AND mirna) OR (plasma AND tumor AND mirna)) AND ('stomach cancer' OR 'gastric cancer' OR 'stomach neoplasm' OR 'gastric neoplasm' OR 'stomach tumor' OR 'gastric tumor') AND ('prognosis' OR 'hazard ratio' OR 'survival')

**Clinicaltrial.gov:** gastric tumor/stomach cancer/stomach tumor | Gastric Cancer | circulating tumor cells

OR gastric tumor/stomach cancer/stomach tumor | Gastric Cancer | circulating tumor DNA/ctDNA/blood ctDNA/serum ctDNA/ plasma ctDNA/ cell-free DNA

OR gastric tumor/stomach cancer/stomach tumor | Gastric Cancer | circulating microRNA/blood miRNA/circulating miRNA/serum miRNA/plasma miRNA

Table S2. Cochrane Risk of bias tool assessment.

| | Were adequate eligibility Criteria developed and applied? | Was the measurement of both exposure and outcome adequate? | Was confounding adequately controlled for? | Was the follow-up complete and adequate in duration? | Are reports of the study free of suggestion of selective outcome reporting? | Was the study free of other problems that put it at a high risk of bias? | Risk of bias? |
|---|---|---|---|---|---|---|---|
| K Hiraiwa | Yes | Yes | Yes | Yes | Yes | Yes | Low |
| S Matsusaka | Yes | Yes | Yes | No | Yes | Yes | High |
| Y Uenosono | Yes | Yes | Yes | Yes | Yes | Yes | Low |
| YL Li | Yes | Yes | Yes | Yes | Yes | Yes | Low |
| H Okabe | Yes | No | Unclear | Yes | Yes | Yes | High |
| SM Yie | Yes | Yes | Yes | Yes | No | Yes | High |
| L Bertazza | Yes | Yes | Yes | Yes | Yes | Yes | Low |
| WG Cao | No | Yes | Yes | No | Yes | Yes | High |
| T Majima | Yes | Yes | Yes | Yes | Yes | Yes | Low |
| AP Noworolska | Yes | Yes | Yes | Yes | Yes | Yes | Low |
| B Illert | Yes | Yes | Yes | Yes | No | Yes | High |
| A Saad | Yes | Yes | Yes | Yes | Yes | Yes | Low |
| YP Liu | Yes | Yes | Yes | Yes | Yes | Yes | Low |
| YH Uen | No | Yes | No | Yes | Yes | Yes | High |
| CH Wu | Yes | Yes | Yes | Yes | Yes | Yes | Low |
| M Ikeguchi | Yes | No | Yes | Yes | Yes | Yes | High |
| S Ishigami | Yes | Yes | No | Yes | Yes | Yes | High |
| MZ Qiu | Yes | Yes | Yes | Yes | Yes | Yes | Low |
| T Arigami | Yes | Yes | Yes | Yes | Yes | Yes | Low |
| H Ito | Yes | Yes | Yes | Yes | Yes | Yes | Low |
| WK Leung | Yes | Yes | No | Yes | Yes | Yes | High |
| B Ioanna | Yes | Yes | Yes | Yes | Yes | Yes | Low |
| QF Yang | Yes | Yes | No | Yes | Yes | Yes | High |
| QL Zhi | Yes | Yes | Yes | Yes | Yes | Yes | Low |
| J Han | Yes | Yes | Yes | Yes | Yes | Yes | Low |
| YC Wu | Yes | Yes | Yes | Yes | Yes | Yes | Low |
| JL Yu | Yes | Yes | Yes | Yes | Yes | Yes | Low |
| B Ioanna | Yes | Yes | Yes | Yes | Yes | Yes | Low |
| P Charinya | No | Yes | No | Yes | Yes | Yes | High |
| WL Fang | Yes | Yes | Yes | Yes | Yes | Yes | Low |
| MV Ayerbes | Yes | Yes | Yes | Yes | Yes | Yes | Low |
| HP Zhang | Yes | Yes | Yes | Yes | Yes | Yes | Low |
| RR Yang | No | Yes | Yes | Yes | No | Yes | High |
| M Wang | Yes | Yes | Yes | Yes | Yes | Yes | Low |

| | | | | | | | |
|---|---|---|---|---|---|---|---|
| S Komatsu | Yes | Yes | Yes | Yes | Yes | Yes | Low |
| JN Song | Yes | Yes | Yes | Yes | Yes | Yes | Low |
| CG Hou | Yes | Yes | Yes | Yes | Yes | Yes | Low |
| H Imaoka | Yes | Yes | Yes | Yes | Yes | Yes | Low |
| ZC Fu | Yes | Yes | Yes | Yes | Yes | Yes | Low |
| DZ Huang | Yes | Yes | Yes | Yes | Yes | Yes | Low |
| Kun Zhuang | Yes | Yes | Yes | Yes | Yes | Yes | Low |
| MM Tsai | Yes | Yes | Yes | Yes | Yes | Yes | Low |
| QJ Chen | Yes | Yes | Yes | Yes | Yes | Yes | Low |

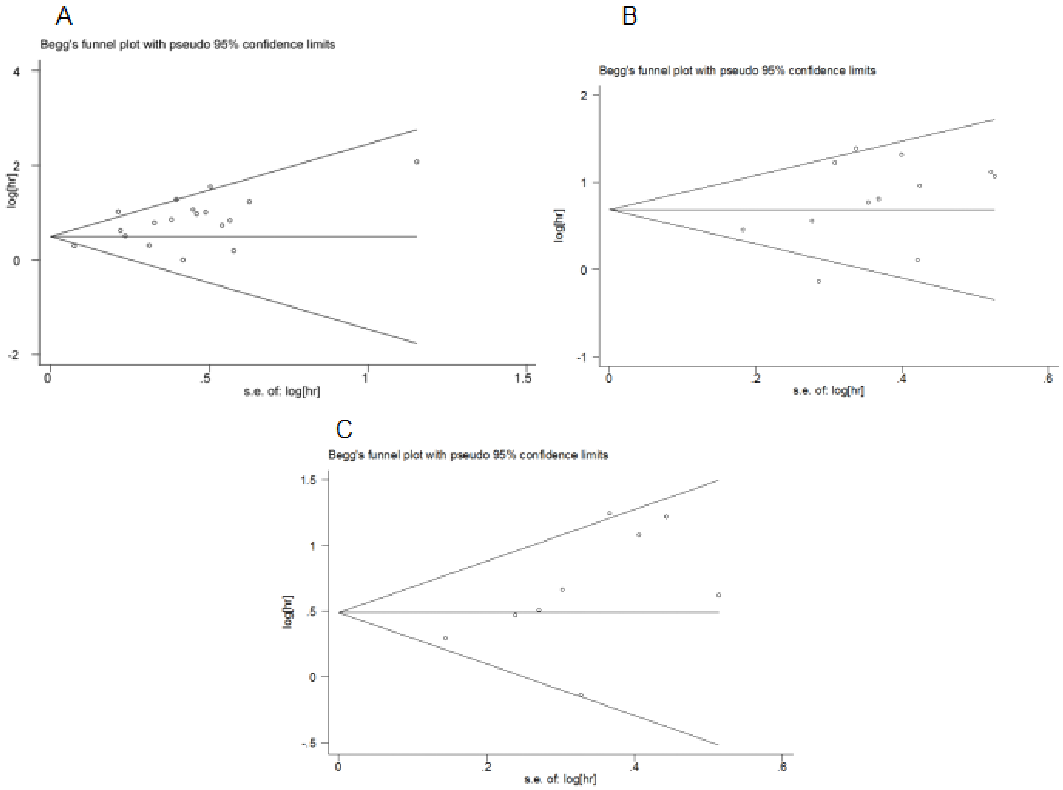


Figure S1 Publication bias by Begg's test (A: CTCs on OS; B: Circulating miRNA on OS; C: ctDNA on OS)

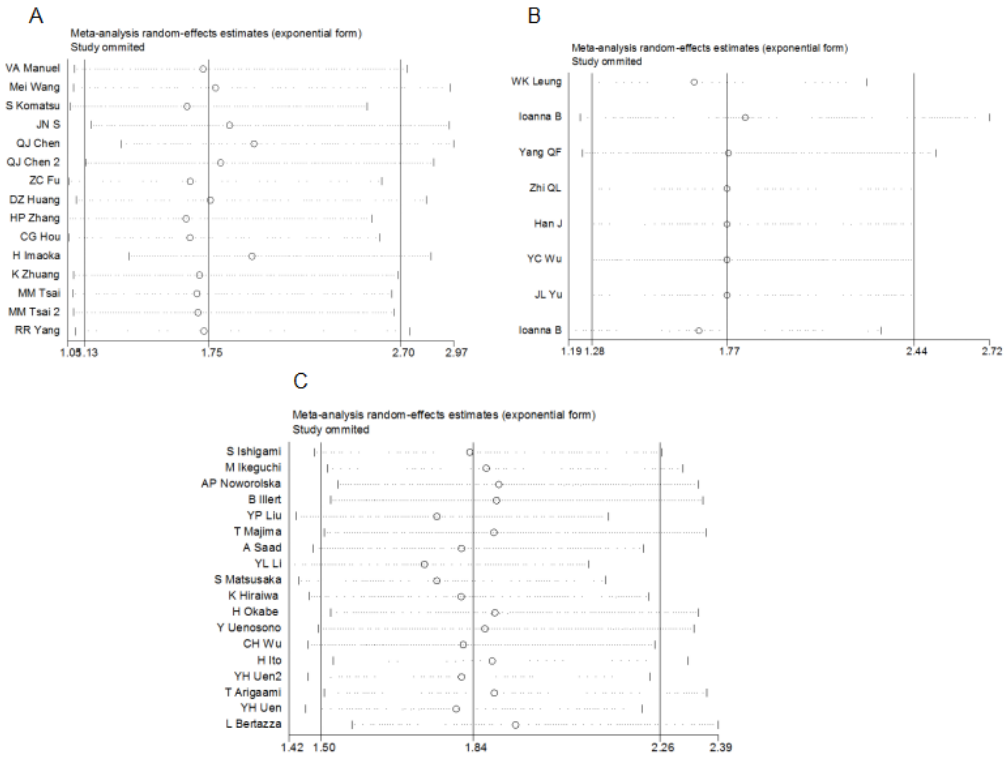


Figure S2. Figure S2 Sensitivity analysis of CTCs (A)/circulating miRNA (B)/ctDNAs (C) in association with GC patients' OS.

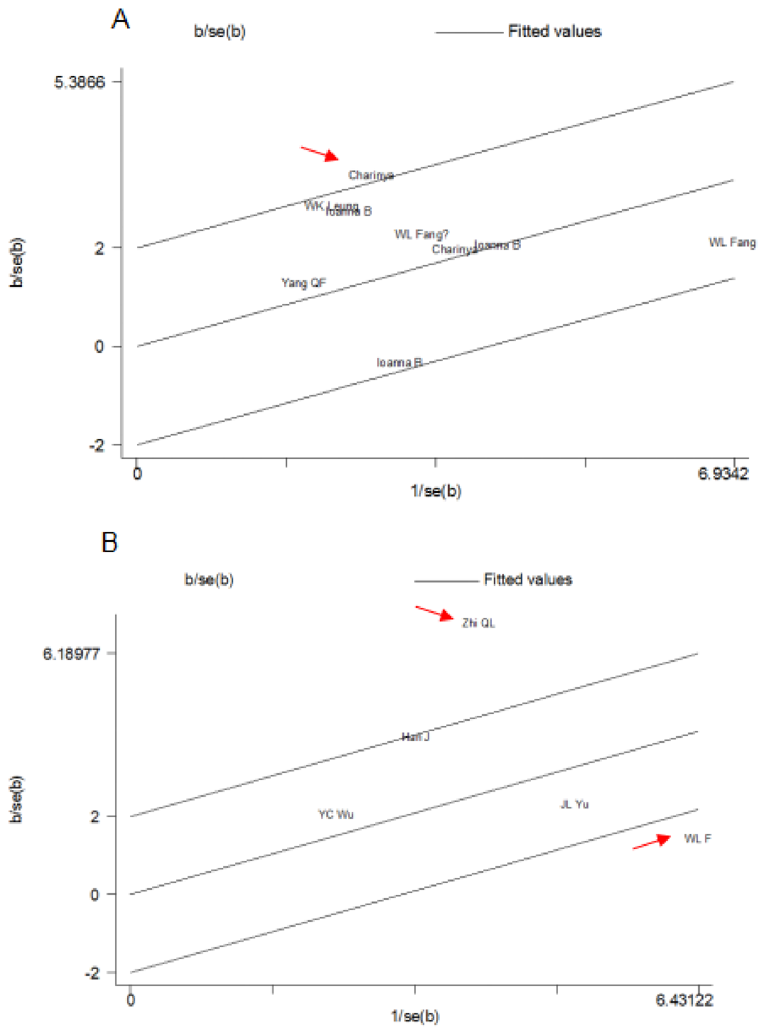


Figure S3. Galbraith plot of ctDNA's association with OS (A) and DFS/PFS (B) respectively.
